# Intrachromosomal amplification 21: A driver of acute myeloid leukemia?

**DOI:** 10.1002/jha2.730

**Published:** 2023-06-04

**Authors:** Nada Assaf, Zaher Chakhachiro

**Affiliations:** ^1^ Department of Pathology and Laboratory Medicine American University of Beirut Medical Center Beirut Lebanon

**Keywords:** acute myeloid leukemia, cancer cytogenetics, chromosomal rearrangements

1

A 68‐year‐old woman was referred for anemia and circulating peripheral blood blasts. Bone marrow aspirate confirmed the diagnosis of acute myeloid leukemia (AML) with a background of variable dysplasia that was not otherwise sufficient to subclassify as AML with myelodysplasia‐related changes (AML‐MRC) (panel A; original magnification × 1000; Wright‐Giemsa stain). By flow cytometry, the blasts were positive for dim/partial CD7, CD13, CD34, CD117, HLA‐DR, partial MPO and TdT, and were negative for other B/T cell associated antigens. G‐banding karyotype showed the presence of tandem duplication at the long arm of chromosome 21 (panel B; 46,XX,dup(21)(q21q22)). Single color fluorescence in situ hybridization probes targeting the 21q22.13‐q22.2 region showed a homogeneously staining region on chromosome 21 containing >5 copies of *RUNX1* (panels C and D; metaphase‐interphase FISH), further confirmed by comparative genomic hybridization. Intrachromosomal amplification 21 (iamp21) is a well‐recognized event in B lymphoblastic leukemia; however, it is rare in AML with <20 published cases, variably associated with morphologic dysplasia although it uniformly occurred in the setting of complex karyotypes. It was thus postulated to be a late event resulting from genome instability and chromothripsis. In our case, iAMP21 occurred as a sole abnormality, suggesting the possibility of a rather early role in leukemogenesis.


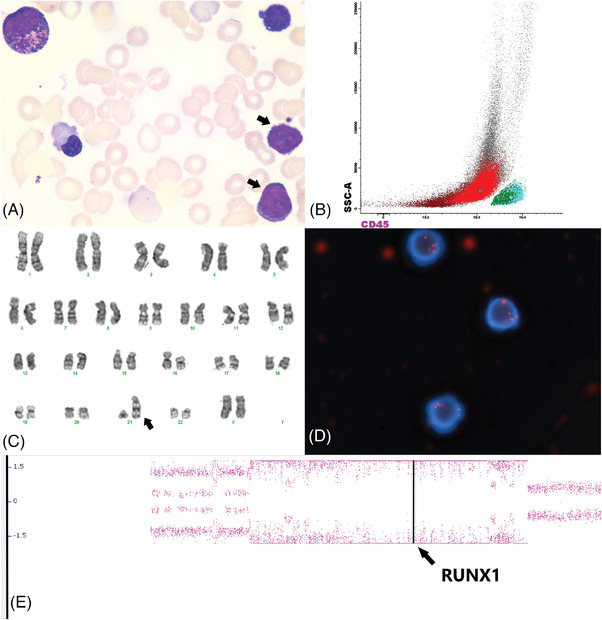


## AUTHOR CONTRIBUTIONS

N.A wrote the manuscript and compiled the genetics images. Z.C designed the cytology and flow cytometry images. Z.C revised the manuscript.

### FUNDING INFORMATION

The study received no funding from internal or external sources.

### CONFLICT OF INTEREST STATEMENT

The authors disclose no conflict of interests.

## ETHICS STATEMENT

The Institutional Review Board at the American University of Beirut exempted our single case report from review. Informed consent was explained to and signed by the patient.

## Data Availability

Data sharing is not applicable to this article as no new data were created or analyzed in this study.

